# Long Non-coding RNA DLEU1 Promotes Cell Proliferation, Invasion, and Confers Cisplatin Resistance in Bladder Cancer by Regulating the miR-99b/HS3ST3B1 Axis

**DOI:** 10.3389/fgene.2019.00280

**Published:** 2019-03-29

**Authors:** Yongzhi Li, Benkang Shi, Fengming Dong, Xingwang Zhu, Bing Liu, Yili Liu

**Affiliations:** ^1^Department of Urology, The Fourth Affiliated Hospital of China Medical University, Shenyang, China; ^2^Department of Urology, Qilu Hospital of Shandong University, Jinan, China

**Keywords:** long non-coding RNA, DLEU1, bladder cancer, miR-99b, HS3ST3B1

## Abstract

Although accumulating evidence has shown the important function of long non-coding RNAs (lncRNAs) in tumor progression and chemotherapy resistance, the role of lncRNA DLEU1 in regulating proliferation, invasion, and chemoresistance of bladder cancer (BCA) cells remains largely unknown. Here, we found that DLEU1 was upregulated in BLCA tissues and BCA patients with high DLEU1 expression exhibited a shorter survival time. Furthermore, mechanistic analysis and functional assays validated that DLEU1 induced cell proliferation, invasion, and cisplatin resistance of BCA cells by de-repressing the expression of HS3ST3B1 through sponging miR-99b. Low miR-99b and high HS3ST3B1 levels were correlated with worse prognosis in patients with BCA. Ectopic expression of HS3ST3B1 or inhibition of miR-99b reversed DLEU1 knockdown-mediated suppression of cell proliferation, invasion, and cisplatin resistance. Thus, our study revealed a novel role for the DLEU1/miR-99b/HS3ST3B1 axis in regulating proliferation, invasion, and cisplatin resistance of BCA cells.

## Introduction

Bladder cancer (BCA) is one of the most common urological malignancies, with an estimated 76,960 new cases and 16,390 deaths in the United States in 2016 (Siegel et al., 2016). Despite advances in the diagnosis and multimodal treatments (including surgery, chemotherapy and radiotherapy), the 5-year survival rate for patients with advanced BCA remains ∼50% (Mohammed et al., 2016). Although cisplatin is commonly used to treat BCA (Dasari and Tchounwou, 2014), the emergence of drug resistance can significantly limit their long-term effectiveness (Chen et al., 2019). The molecular mechanisms underlying BCA progression and chemoresistance are not well understood.

An increasing number of studies have reported that the dysregulation of microRNAs (miRNAs) and long non-coding RNAs (lncRNAs) can regulate the progression and chemoresistance of human tumors (Anastasiadou et al., 2018). miRNAs are small, endogenous non-coding coding RNAs that post-transcriptionally modulate gene expression by binding to complementary sequences on particular messenger RNA transcripts (Anastasiadou et al., 2018). lncRNAs are RNA molecules of greater than 200 nucleotides and can act as molecular sponges, scaffolds, and guides to interact with proteins, mRNAs, or miRNAs, thereby regulating the expression of target genes (Anastasiadou et al., 2018; Dong et al., 2018). Growing evidence indicated that the interactions between lncRNAs and miRNAs play crucial roles in modulating tumor progression and chemoresistance (Cao et al., 2017; Chen et al., 2019).

Long non-coding RNA DLEU1 is overexpressed and promotes migration and invasion in ovarian cancer (Wang et al., 2017), colorectal cancer (Liu et al., 2018), pancreatic cancer (Gao et al., 2018), and lung cancer (Zhang et al., 2019). In this study, we investigated the role of DLEU1 in regulating proliferation, invasion and cisplatin resistance in BCA cells. We found that the expression of DLEU1 was upregulated in BCA tissues compared with normal tissues. The overexpression of DLEU1 predicted poor prognosis of patients with BCA. DLEU1 promoted the proliferation and invasion and conferred resistance to cisplatin by upregulating the expression of oncogene HS3ST3B1 via suppressing miR-99b, a direct inhibitor of HS3ST3B1 in BCA cells. Our findings have demonstrated for the first time that DLEU1 possesses a crucial role in proliferation, invasion, and cisplatin resistance in BCA cells through regulation of the miR-99b/HS3ST3B1 pathway.

## Materials and Methods

### Tissue Samples

Twenty pairs of BCA and adjacent normal tissues were collected from patients with primary BCA at the Fourth Affiliated Hospital of China Medical University, China. None of these patients received chemotherapy or radiotherapy. Samples were immediately frozen in liquid nitrogen and stored at −80°C until used for RNA extraction. Our study was approved by the Research Ethics Committee of Fourth Affiliated Hospital of China Medical University and written informed consents were obtained under the agreement of all patients with pathological confirmation.

### Cell Lines, Culture Conditions, and Cell Transfection

Human BCA cell lines (T24 and SW780) were purchased from the Shanghai Institute of Cell Biology (Shanghai, China). Primary normal human bladder epithelial cells (normal cells) were obtained from The American Type Culture Collection. These cells were maintained in RPMI-1640 medium (Invitrogen, Carlsbad, CA, United States) supplemented with 5% fetal bovine serum (FBS, Gibco, Grand Island, NY, United States) at 37°C with 5% of CO_2_ in a humidified atmosphere. Two different siRNAs against DLEU1 (siRNA-1/2), control siRNA, miR-99b mimic, miR-99b inhibitor and their respective controls were obtained from RiboBio (Guangzhou, China). The DLEU1 expression vector and the HS3ST3B1 expression vector were constructed by Genepharma (Shanghai, China). Cells were transfected using Lipofectamine 2000 reagent (Invitrogen, CA, United States) following the manufacturer’s protocol.

### RNA Extraction and Real-Time PCR Analysis (qRT-PCR)

Total RNA was isolated from BCA samples and cells using TRIzol reagent (Invitrogen, Carlsbad, CA, United States). Sample RNA (1 μg) was reverse transcribed using a Reverse Transcription Kit (Takara, Dalian, China). Quantitative real-time PCR was performed using an SYBR Green Real-time PCR Master Mix kit (Toyobo, Osaka, Japan) on the 7900HT fast real-time PCR system (Applied Biosystems, San Francisco, CA, United States). The levels of miR-99b were quantified with the mirVana^TM^ qRT-PCR microRNA Detection Kit (Ambion, Austin, TX, United States). The primers used in this study were synthesized by Genepharma (Shanghai, China). The primer sequences for qRT-PCR were purchase from Genepharma (Shanghai, China). The results were normalized to the levels of *GAPDH* or U6, respectively.

### Western Blotting Analysis

Cells were lysed with ice-cold RIPA lysis buffer (Solarbio, Beijing, China) containing Protease Inhibitor Cocktail (Roche, Shanghai, China). Equal amount of protein lysates were separated by 10% SDS-polyacrylamide gel electrophoresis and transferred on polyvinylidene difluoride membrane (Millipore, Bedford, MA, United States) and probed by antibodies against HS3ST3B1 (Sigma-Aldrich Co., St. Louis, MO, United States, 1:1000) and GAPDH (Santa Cruz, CA, United States, 1:5000). Following incubation with the corresponding secondary antibodies, signals were detected with the ECL detection kit (Pierce, Rockford, IL, United States).

### CCK-8 Assay

Five thousand cells were seeded into 96-well plates and transfected with DLEU1 siRNA, control sRNA, DLEU1 expression vector or control vector for 72 h. Cell viability was measured using the CCK-8 (Dojindo, Kumamoto, Japan) according to the manufacturer’s instructions.

### Colony Formation Assay

Colony formation assay was conducted as reported (Ihira et al., 2017).

### Apoptosis Assay

Three thousand cells were seeded in 96-well plates and transfected as indicated. After 24 h of incubation, cells were treated with saline or varying doses of cisplatin (Sigma-Aldrich Co., St. Louis, MO, United States). After 24 h of treatment, cell viability was determined using the CCK-8 (Dojindo, Kumamoto, Japan). Values obtained were expressed as the percentage of surviving cells, with the viability of saline-treated cells set at 100%. Cell apoptosis was measured by the Caspase-Glo 3/7 assay reagent (Promega, Madison, WI, United States) as described (Dong et al., 2016).

### Cell Invasion Assay

Transwell cell invasion assays were performed using Boyden chambers (Corning, New York, NY, United States) that use 8 μm pore membranes with Matrigel as reported (Xiong et al., 2017). In brief, 1 × 10^5^ cells were added to a Matrigel invasion chamber and FBS was added to the lower chamber. After 24 h, the non-invading cells were gently removed with a cotton swab. Invaded cells were stained with 1% toluidine blue solution and counted.

### Luciferase Reporter Assay

The fragment of DLEU1 or HS3ST3B1 3′-UTR (wild-type: WT; mutant: MUT) containing the miR-99b binding site was synthesized and then cloned into the pGL3-basic vector (Promega, Madison, WI, United States). Cells were seeded into 24-well plates. Each luciferase reporter vector was co-transfected with pRL-CMV (Promega, Madison, WI, United States) expressing Renilla luciferase, and miR-99b mimic, miR-99b inhibitor or their respective controls using Lipofectamine 2000 reagent (Invitrogen, CA, United States). After 48 h, cell lysates were made. Firefly and Renilla luciferase activities were measured using the Dual-Luciferase Reporter Assay System (Promega, Madison, WI, United States) according to the manufacturer’s instructions. Firefly luciferase activity was normalized to that of Renilla luciferase activity for each sample.

### RNA Immunoprecipitation Assay (RIP)

The RIP assay was performed with the Magna RIP RNA-Binding Protein Immunoprecipitation Kit (Millipore, Bedford, MA, United States) following the manufacturer’s protocol. Briefly, cells were collected and lysed using RIP lysis buffer. One hundred microliters of cell extract was incubated with RIP buffer containing magnetic beads conjugated to an anti-Argonaute2 (Ago2) antibody (Millipore, Bedford, MA, United States) or negative control IgG (Millipore, Bedford, MA, United States). The samples were incubated with Proteinase K to digest proteins and then the immunoprecipitated RNA was isolated. The purified RNA was subjected to quantitative PCR to detect the presence of DLEU1 or miR-99b. The total RNAs were the input controls.

### Statistical Analysis

The results are the means ± standard deviation from at least three experiments. Statistical evaluations were carried out with SPSS 22.0 software (Chicago, IL, United States). Differences between two groups were compared using Student’s *t*-test, and differences among three groups were analyzed by ANOVA. *P*-values <0.05 were considered significant.

## Results

### lncRNA-DLEU1 Is Elevated in BCA Tissues and High DLEU1 Expression Predicts Poor Prognosis of BCA Patients

**FIGURE 1:**
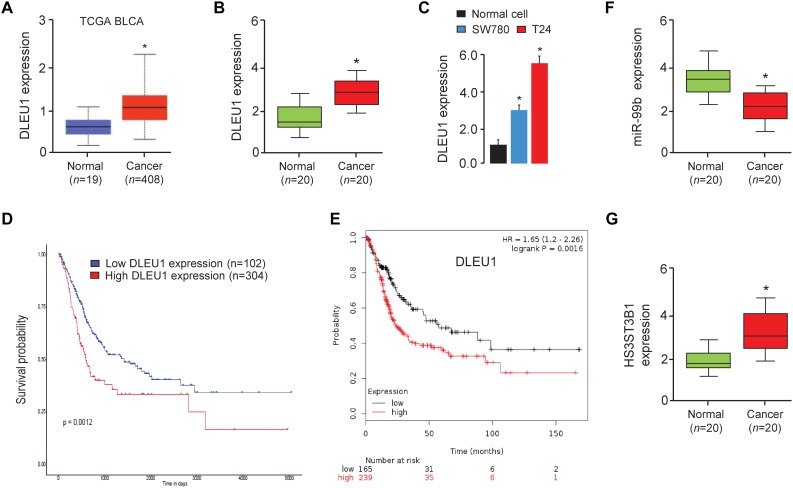
DLEU1 is upregulated in BCA tissues and high DLEU1 expression predicts poor disease prognosis. **(A)** The expression of DLEU1 in normal and BCA tissues from the TCGA database UALCAN. **(B)** qRT-PCR analysis of DLEU1 expression in BCA samples (*n* = 20) and adjacent normal tissues (*n* = 20). **(C)** qRT-PCR analysis of DLEU1 expression in BCA cell lines and normal cells. **(D,E)** High expression of DLEU1 decreases survival probability. The publicly available TCGA datasets were obtained from the web portal of UALCAN **(D)** and KMplotter **(E)**. **(F)** qRT-PCR analysis of miR-99b expression in BCA samples (*n* = 20) and adjacent normal tissues (*n* = 20). **(G)** qRT-PCR analysis of HS3ST3B1 expression in BCA samples (*n* = 20) and adjacent normal tissues (*n* = 20). ^∗^*P* < 0.05.

In order to investigate the relevance of DLEU1 in BCA development, we first checked its expression using the TCGA BCA dataset through the UALCAN web portal (Chandrashekar et al., 2017). As shown in Figure 1A, the expression of DLEU1 in BCA tissues was significantly higher than that in normal tissues. Then, the expression levels of DLEU1 were examined in 20 pairs of BCA tissues and adjacent normal tissues. Our qRT-PCR analysis demonstrated that DLEU1 was upregulated in BCA samples compared to adjacent normal tissues (Figure 1B). The expression of DLEU1 was measured in two BCA cell lines (SW780 and T24) and normal cells. Higher levels of DLEU1 were detected in SW780 and T24 cells as compared with normal cells (Figure 1C). Through the web tools, including UALCAN and the KMplotter (Győrffy et al., 2013), we found that higher levels of DLEU1 were positively correlated with reduced patient survival (Figure 1D,E). These findings demonstrated that DLEUU1 may play an important role in the regulation of BCA development and may serve as a prognostic marker in BCA.

### DLEU1 Acts as an Oncogene by Promoting the Proliferation and Invasion of BCA Cells

**FIGURE 2:**
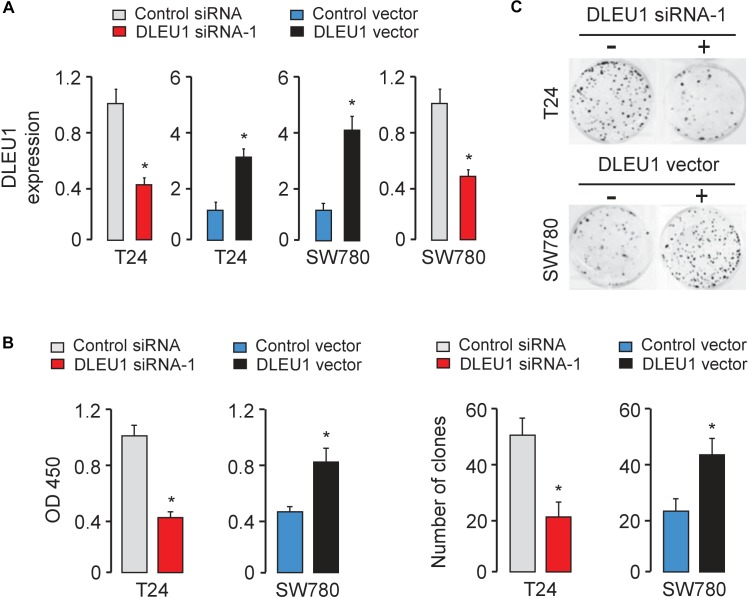
Knockdown of DLEU1 inhibits BCA cell proliferation and invasion *in vitro*. **(A)** Validation of the transfection efficiency in BCA cell lines using qRT-PCR analysis. **(B)** DLEU1 induces cell proliferation as measured by CCK-8 assays in T24 and SW780 cells. **(C)** DLEU1 promotes colony formation as demonstrated by colony formation assays in T24 and SW780 cells. ^∗^*P* < 0.05.

Next, we explored the function of DLEU1 in tumorigenesis and development of BCA. Because T24 cells have relatively high expressions of DLEU1, whereas SW780 cells have relatively low expressions of DLEU1 (Figure 1B), we silenced the expression of endogenous DLEU1 in T24 cells using two different siRNAs, and overexpressed DLEU1 in SW780 cells by using an expression vector containing human DLEU1. In addition, we also overexpressed DLEU1 in T24 cells and silenced the expression of DLEU1 in SW780 cells. The qRT-PCR analysis was used to confirm the transfection efficiency (Figure 2A and Supplementary Figure 1A).

**FIGURE 3:**
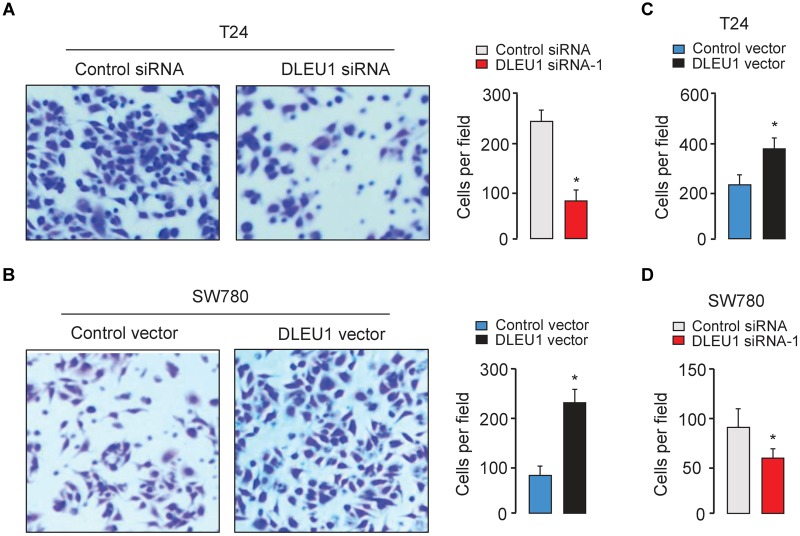
DLEU1 promotes cell invasion in BCA cells. Representative images (left) and bar graphs (right) depicting the invasion ability of T24 **(A)** and SW780 **(B)** cells after the knockdown or overexpression of DLEU1, and of T24 cells **(C)** and SW780 cells after the overexpression or knockdown of DLEU1 **(D)**. ^∗^*P* < 0.05.

The CCK-8 and colony formation assay demonstrated that the knockdown of DLEU1 attenuated the proliferation of T24 cells, and overexpression of DLEU1 significantly enhanced the proliferation of SW780 cells (Figure 2B,C and Supplementary Figure 1B). We examined cell invasion by transwell assay after the knockdown or overexpression of DLEU1. We observed that the invasive abilities of T24 cells were remarkably reduced upon silencing of DLEU1 compared with the control cells (Figure 3A and Supplementary Figure 1C). Meanwhile, the invasion of SW780 cells was significantly induced following upregulation of DLEU1 expression as shown by transwell assay (Figure 3B). Moreover, the upregulation of DLEU1 in T24 cells enhanced cell invasion, and downregulation of DLEU1 in SW780 cells decreased cell invasion (Figure 3C,D). Taken together, these findings indicated the growth and invasion-stimulating roles of DLEU1 in BCA.

### DLEU1 Confers Resistance to Cisplatin-Induced Apoptosis

**FIGURE 4:**
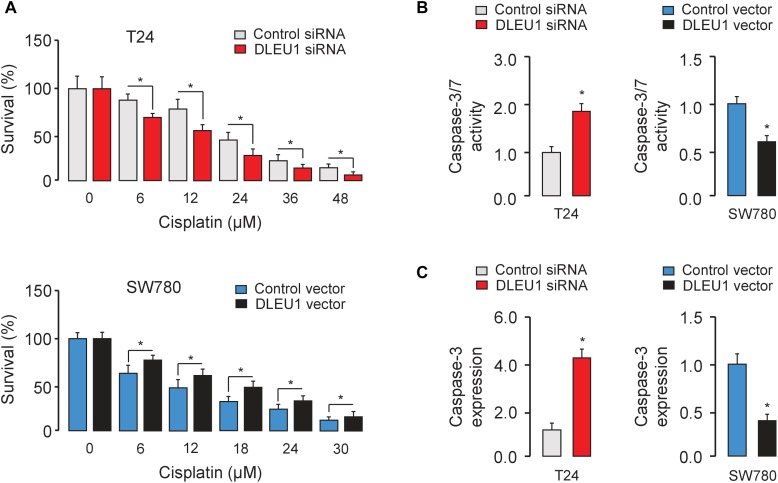
Knockdown of DLEU1 enhances the sensitivity of BCA cells to cisplatin-induced apoptosis. **(A)** T24 (upper panel) and SW780 (bottom panel) cells were transfected as indicated for 24 h, and then cells were incubated with or without cisplatin for another 24 h. Cell viability was detected using CCK-8 assays. **(B)** The effects of cisplatin on apoptosis of BCA cells were measured using Caspase-Glo 3/7 assays. **(C)** qRT-PCR analysis of caspase-3 expression in T24 and SW780 cells after the knockdown or overexpression of DLEU1. ^∗^*P* < 0.05.

Next, we evaluated the effect of DLEU1 on cisplatin sensitivity in BCA cells. Following the knockdown or overexpression of DLEU1, BCA cells were treated with cisplatin and then assayed for their sensitivity to cisplatin using the CCK-8 assay. Our results showed that DLEU1 silencing increased the sensitivity to cisplatin (Figure 4A) and enhanced the cisplatin-induced cell apoptosis, as determined by an increase in the activities of caspase-3/7 in T24 cells (Figure 4B). Conversely, ectopic overexpression of DLEU1 endowed SW780 cells with refractoriness to cisplatin (Figure 4A) and antagonized cisplatin-induced apoptosis (Figure 4B). We next explored whether the modulation of DLEU1 alter the expression of caspase-3 in BCA cells using qRT-PCR analysis. Our results showed that the levels of caspase-3 was significantly increased after the knockdown of DLEU1 in T24 cells, while was significantly repressed after the overexpression of DLEU1 in SW780 cells (Figure 4C). Consequently, these results indicated the possibility that the knockdown of DLEU1 sensitized BCA cells to cisplatin-induced apoptosis.

### DLEU1 Sponges miR-99b and Indirectly Upregulates HS3ST3B1 Expression

**FIGURE 5:**
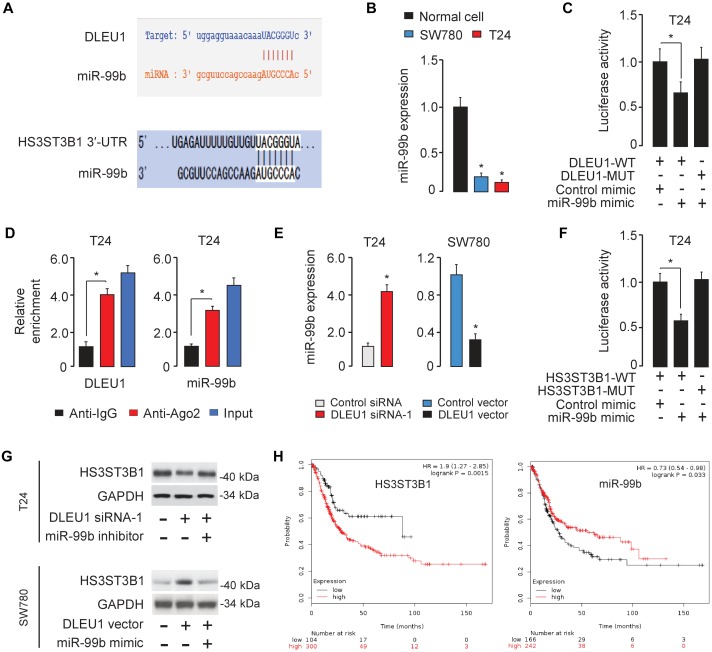
DLEU1 sponges miR-99b to upregulate HS3ST3B1 expression. **(A)** The miR-99b-binding sequences in DLEU1 and HS3ST3B1 3′-UTR. **(B)** qRT-PCR analysis of miR-99b expression in BCA cell lines and normal cells. **(C)** Luciferase reporter assay in T24 cells, co-transfected with a luciferase reporter plasmid containing wild-type (WT) or mutant (MUT) DLEU1 and the indicated miRNAs. **(D)** RIP assay showed the enrichment of DLEU1 and miR-499b in Ago2-containing beads. **(E)** qRT-PCR analysis of miR-99b expression in BCA cells after the knockdown or overexpression of DLEU1. **(F)** Luciferase reporter assay in T24 cells, co-transfected with a luciferase reporter plasmid containing wild-type (WT) or mutant (MUT) HS3ST3B1 3′-UTR and the indicated miRNAs. **(G)** The protein expression of HS3ST3B1 in T24 cells co-transfected with DLEU1 siRNA or control siRNA, together with miR-99b inhibitor or its respective control were analyzed using western blotting analysis (upper panel). The protein expression of HS3ST3B1 in SW780 cells co-transfected with DLEU1 expression vector or control vector, together with miR-99b mimic or its respective control were examined using western blotting analysis (bottom panel). **(H)** Kaplan–Meier survival analysis of overall survival in BCA patients based on HS3ST3B1 or miR-99b expression. The publicly available TCGA datasets were obtained from the web portal of KMplotter. ^∗^*P* < 0.05.

Long non-coding RNAs can function as competing endogenous RNAs to modulate the expression and biological functions of miRNAs. Thus, we speculated that DLEU1 might promote BCA tumorigenesis, progression and cisplatin resistance by suppressing the functions of certain miRNAs. The potential miRNA targets of DLEU1 were predicted by the bioinformatics database StarBase V2.0 (Li et al., 2014). We identified the miR-99b-binding site in DLEU1 (Figure 5A). Interestingly, the expression level of miR-99b was significantly reduced in BCA samples compared to normal samples (Figure 1F), and BCA cell lines had lower levels of miR-99b than the normal cells (Figure 5B). These data suggested that the expression of miR-99b was negatively correlated with that of DLEU1 in BCA cells.

To further explore the relationship between DLEU1 and miR-99b, a dual-luciferase reporter assay was performed. The transfection with miR-99b mimic significantly reduced the luciferase activity of wild-type DLEU1 reporter vector, but not that of mutant reporter vector in T24 cells (Figure 5C), suggesting that DLEU1 was directly targeted by miR-99b in BCA cells. In order to further verify whether DLEU1 and miR-99b are in the same RNA-induced silencing complex, anti-Ago2 RIP assay was performed in T24 cells. The expression level of DLEU1 and miR-99b was higher in the anti-Ago2 group than that in the anti-normal IgG group (Figure 5D). Importantly, the knockdown of DLEU1 elevated the levels of miR-99b, and the overexpression of DLEU1 reduced the expression levels of miR-99b in T24 cells (Figure 5E), indicating that DLEU1 and miR-99b mutually regulate each other. Taken together, the results suggested that DLEU1 interacts with miR-99b and regulates its expression.

We further searched for the target genes of miR-99b using a bioinformatics program TargetScan, and found that *HS3ST3B1* mRNA contained a potential target site of miR-99b in its 3′-UTR (Figure 5A). Previous studies have shown that HS3ST3B1 was upregulated in several tumors and can maintain the mesenchymal phenotype and promote cancer cell proliferation and angiogenesis (Song et al., 2011; Zhang et al., 2015, 2018; Hellec et al., 2018). Our qRT-PCR analysis showed that HS3ST3B1 expression was significantly upregulated in BCA samples compared to adjacent normal samples (Figure 1G). Dual-luciferase reporter assays demonstrated that the transfection with miR-99b mimic attenuated the luciferase activities of the wild-type HS3ST3B1 3′-UTR, but had no effect on the mutant 3′-UTR of HS3ST3B1 (Figure 5F), verifying the direct binding between miR-99b and HS3ST3B1 in BCA cells.

Furthermore, the protein levels of HS3ST3B1 in T24 cells were reduced after transfecting with DLEU1 siRNA-1 (Figure 5G). This inhibitory effect of DLEU1 silencing was notably reversed by co-transfection with miR-99b inhibitor in T24 cells (Figure 5G). In SW780 cells, the expression of HS3ST3B1 was significantly increased after transfecting with the DLEU1 expression vector (Figure 5G). This increase in HS3ST3B1 expression was reversed by co-transfection with miR-99b mimic (Figure 5G). The relationship between HS3ST3B1 or miR-99b expression and patient survival was analyzed in a TCGA BCA cohort using the KMplotter web tool. We found that higher HS3ST3B1 levels or lower miR-99b levels were significantly correlated with decreased overall survival (Figure 5H). These results suggested that DLEU1 functions as a competing endogenous RNA by sponging miR-99b and indirectly upregulated HS3ST3B1 expression in BCA cells.

### DLEU1 Promotes Proliferation, Invasion, and Chemoresistance by Upregulating HS3ST3B1 Expression Through Protecting It From miR-99b-Mediated Suppression

**FIGURE 6:**
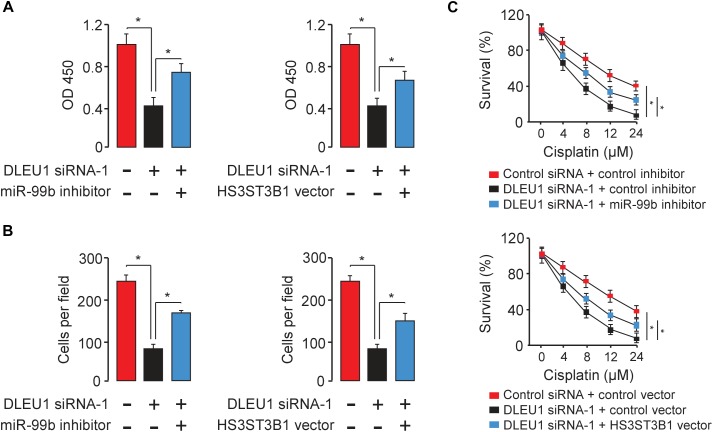
DLEU1 promotes BCA cell proliferation, invasion, and cisplatin resistance by sponging miR-99b and restoring HS3ST3B1 expression. Decreased cell proliferation **(A)**, invasion **(B)**, and cisplatin resistance **(C)** upon DLEU1 knockdown in T24 cells were rescued by miR-99b silencing or ectopic expression of HS3ST3B1. ^∗^*P* < 0.05.

To test whether DLEU1 exerts oncogenic functions in BCA by modulating the miR-99b/HS3ST3B1 axis, we investigated the effects of miR-99b or HS3ST3B1 on DLEU1-induced cancer cell proliferation, invasion, and cisplatin resistance. We observed that the knockdown of the endogenous DLEU1 significantly weakened the proliferation, invasion and enhanced the sensitivity of T24 cells to cisplatin treatment (Figure 6). These influences of DLEU1 knockdown were reversed by down-regulation of miR-99b or ectopic expression of HS3ST3B1 in T24 cells (Figure 6). Collectively, these data supported the hypothesis that DLEU1 promotes BCA cell proliferation, invasion, and cisplatin resistance, at least partly, through sponging miR-99b and then upregulating HS3ST3B1 expression.

## Discussion

Long non-coding RNAs have important roles in various types of malignant tumors, and some lncRNAs may function as novel diagnostic markers and therapeutic targets in BCA (Quan et al., 2018; Taheri et al., 2018; Wieczorek and Reszka, 2018). DLEU1 is aberrantly overexpressed in a variety of tumors and contributes to tumorigenesis and cancer development (Wang et al., 2017; Gao et al., 2018; Liu et al., 2018; Zhang et al., 2019). In this study, we demonstrated for the first time that, the expression of DLEU1 is significantly increased in BCA tissues and BCA cell lines, and elevated expression of DLEU1 predicts worse patient survival. Subsequent functional investigation revealed that DLEU1 promotes BCA cell growth, invasion and induces cisplatin resistance. Furthermore, we showed that DLEU1 executes its tumor-promoting functions by sponging tumor suppressor miR-99b and increasing the protein expression of oncogene HS3ST3B1 in BCA.

To investigate the underlying mechanisms of DLEU1, we have identified miR-99b as a direct target of DLEU1 in BCA cells. Dual-luciferase reporter and RIP assay demonstrated the direct interaction between DLEU1 and miR-99b. Emerging studies have shown that miR-99b was downregulated and served as a novel tumor suppressor in many cancers, including lung cancer (Kang et al., 2012), cervical cancer (Li et al., 2018), colorectal cancer (Li et al., 2015), and gastric cancer (Wang et al., 2018). However, miR-99b was shown to induce cancer cell migration and invasion and metastasis in esophageal squamous cell carcinoma (Ma et al., 2017). Currently, the function of miR-99b in BCA remains unclear. We showed here that the downregulation of miR-99b was associated with poor survival in patients with BCA, and miR-99b can partly reverse DLEU1-induced cell proliferation, invasion, and cisplatin resistance, suggesting that miR-99b has important roles in suppressing BCA progression and overcoming cisplatin resistance in BCA cells.

MicroRNAs could regulate the expression of multiple target genes simultaneously. In the present study, we found that miR-99b bound directly to HS3ST3B1 in BCA cells. HS3ST3B1 was overexpressed in tumors and participated in regulating the invasive phenotype, cell proliferation, and angiogenesis (Song et al., 2011; Zhang et al., 2015, 2018; Hellec et al., 2018), but little was known about its function in BCA. Here, we showed that high HS3ST3B1 levels were correlated with a shorter survival time of BCA patients, and the ectopic expression of HS3ST3B1 could promote the proliferation and invasion of BCA cells, and enhance the resistance of BCA cells to cisplatin treatment. Taken together, these data suggested that HS3ST3B1 has diverse oncogenic roles in promoting the proliferation, invasion and chemoresistance in BCA cells.

## Conclusion

In conclusion, the present study showed that DLEU1 is upregulated in BCA and promotes BCA cell proliferation, invasion, and cisplatin resistance via competitively sponging miR-99b and then restoring HS3ST3B1 expression. Therefore, the DLEU1/miR-99b/HS3ST3B1 axis represents a key pathway involved in BCA progression and cisplatin resistance, and DLEU1 could be considered as a potential target for BCA therapies in the future.

## Data Availability

All data generated during this study are available from the corresponding author on reasonable request.

## Author Contributions

YiL designed the experiments. YoL and BS performed the experiments. FD, XZ, and BL made significant revisions to the manuscript. All authors read and approved the final manuscript.

## Conflict of Interest Statement

The authors declare that the research was conducted in the absence of any commercial or financial relationships that could be construed as a potential conflict of interest.
