# Supplementary material for: Long Non-coding RNA DLEU1 Promotes Cell Proliferation, Invasion, and Confers Cisplatin Resistance in Bladder Cancer by Regulating the miR-99b/HS3ST3B1 Axis
Source: Front Genet. 2019 Mar 29;10:280. doi: 10.3389/fgene.2019.00280 (PMC6449426; doi:10.3389/fgene.2019.00280)

Supplementary Material

Long non-coding RNA DLEU1 promotes cell proliferation, invasion and confers cisplatin resistance in bladder cancer by regulating the miR-99b/HS3ST3B1 axis

Yongzhi Li[1, †], Benkang Shi[2, †], Fengming Dong[1], Xingwang Zhu[1], Bing Liu[1] and Yili Liu[1,*]

*** Correspondence:** Yili Liu: yililiu2010@yahoo.co.jp

Supplementary Figure 1

Figure 1. Knockdown of DLEU1 reduced the proliferation and invasion of T24 cells.

(A). Efficiency of DLEU1 knockdown in T24 cells by DLEU1 siRNA-2 was verified by qRT-PCR analysis. **(B)**. Knockdown of DLEU1 reduced cell proliferation of T24 cells as measured by CCK-8 assays. **(C)**. Knockdown of DLEU1 reduced cell invasion of T24 cells as measured by transwell invasion assays. **P*<0.05.

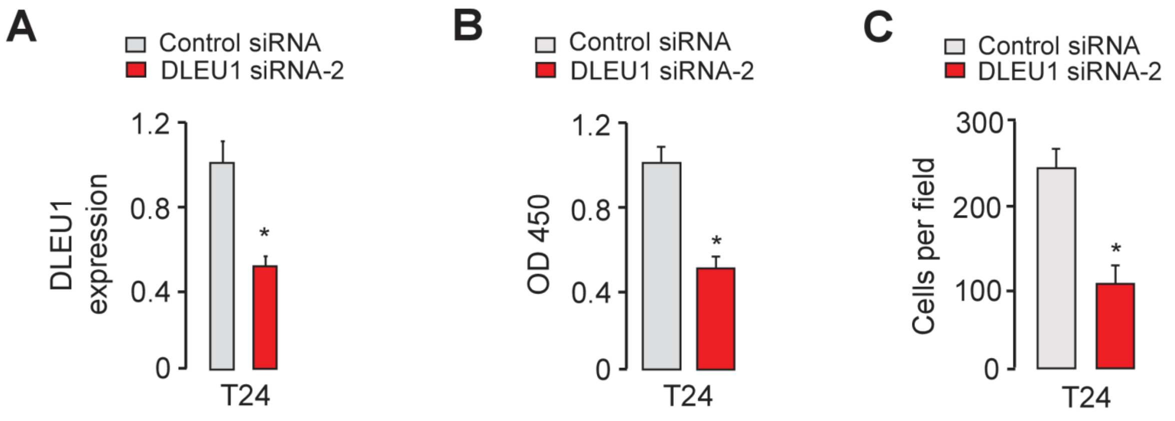

Supplement: Supplementary file 1 [file Image_1.pdf]
